# Supplementary material for: High-resolution genome-wide mapping of chromosome-arm-scale truncations induced by CRISPR–Cas9 editing
Source: Nat Genet. 2024 May 29;56(7):1482–93. doi: 10.1038/s41588-024-01758-y (PMC11250378; doi:10.1038/s41588-024-01758-y)
Supplement: Supplementary file 1 — Reporting Summary [file 41588_2024_1758_MOESM1_ESM.pdf]

nature portfolio

Corresponding author(s): Imran Haque

Last updated by author(s): Mar 7, 2024

Reporting Summary

Nature Portfolio wishes to improve the reproducibility of the work that we publish. This form provides structure for consistency and transparency in reporting. For further information on Nature Portfolio policies, see our Editorial Policies and the Editorial Policy Checklist.

Statistics

For all statistical analyses, confirm that the following items are present in the figure legend, table legend, main text, or Methods section.

n/a	Confirmed	
☐	☒	The exact sample size (*n*) for each experimental group/condition, given as a discrete number and unit of measurement
☐	☒	A statement on whether measurements were taken from distinct samples or whether the same sample was measured repeatedly
☐	☒	The statistical test(s) used AND whether they are one- or two-sided<br>*Only common tests should be described solely by name; describe more complex techniques in the Methods section.*
☒	☐	A description of all covariates tested
☐	☒	A description of any assumptions or corrections, such as tests of normality and adjustment for multiple comparisons
☐	☒	A full description of the statistical parameters including central tendency (e.g. means) or other basic estimates (e.g. regression coefficient) AND variation (e.g. standard deviation) or associated estimates of uncertainty (e.g. confidence intervals)
☐	☒	For null hypothesis testing, the test statistic (e.g. *F*, *t*, *r*) with confidence intervals, effect sizes, degrees of freedom and *P* value noted<br>*Give P values as exact values whenever suitable.*
☒	☐	For Bayesian analysis, information on the choice of priors and Markov chain Monte Carlo settings
☒	☐	For hierarchical and complex designs, identification of the appropriate level for tests and full reporting of outcomes
☐	☒	Estimates of effect sizes (e.g. Cohen's *d*, Pearson's *r*), indicating how they were calculated

Our web collection on statistics for biologists contains articles on many of the points above.

Software and code

Policy information about availability of computer code

Data collection	Data were downloaded and processed with python (v3.9), numpy (v1.22.3) and pandas (1.4.2) and R (v4.1)
Data analysis	Analysis was performed using Python (v3.9) and open-source packages numpy(v1.22.3), pandas(v1.4.2), scipy (v1.10.1) and statsmodels (v0.13.2). Visualizations generated with matplotlib (v3.5.2), scikit-image (v0.18.3), seaborn (v0.12.3). single-cell RNA sequencing data was processed with STAR (v2.7.7a) and scanpy (v1.9.3) and determined the CNV events using infercnvpy (v0.4.1). The list of cancer genes was downloaded from OncoKB (v4.4).<br>Custom code available at https://github.com/recursionpharma/proxbias/

For manuscripts utilizing custom algorithms or software that are central to the research but not yet described in published literature, software must be made available to editors and reviewers. We strongly encourage code deposition in a community repository (e.g. GitHub). See the Nature Portfolio guidelines for submitting code & software for further information.

Data

Policy information about availability of data

All manuscripts must include a data availability statement. This statement should provide the following information, where applicable:

- Accession codes, unique identifiers, or web links for publicly available datasets
- A description of any restrictions on data availability
- For clinical datasets or third party data, please ensure that the statement adheres to our policy

Raw images, metadata, and deep-learning-derived embeddings for rxrx3 are available at https://rxrx.ai. cpg0016 is available as part of the JUMP Cell Painting datasets available from the Cell Painting Gallery at https://registry.opendata.aws/cellpainting-gallery/. DepMap data are available at https://depmap.org/portal/download/all/ JUMP CP data was downloaded from their S3 bucket using python (1.22.3) and pandas (1.4.2): https://registry.opendata.aws/cellpainting-gallery/. hg38 gene locations and annotations were downloaded from UCSC: https://genome.ucsc.edu/cgi-bin/hgTables shinyDepMap data was downloaded and processed using R (4.1): https://depmap.org/portal/download/all/ Files containing scRNAseq AnnData objects for two CRISPR-Cas9 and three CRISPRi screens were downloaded from zenodo.org/record/7416068. The list of cancer genes was downloaded from OncoKB (v4.4), https://www.oncokb.org/cancer-genes.

Research involving human participants, their data, or biological material

Policy information about studies with human participants or human data. See also policy information about sex, gender (identity/presentation), and sexual orientation and race, ethnicity and racism.

Reporting on sex and gender	The HUVEC source cells used in the rxrx3 dataset were sourced from two donor pools.<br>1) Lonza Catalog # C2519A Lot 0000662339 Manufacture Date 26-Sept-2017 (3 pooled umbilical cord donors) Age = Newborn, Sex = Male, Male, Male, Race = Caucasian, Caucasian, Caucasian<br>2) Lonza Catalog # C2519A Lot 0000661173 Manufacture Date 22-Sept-2017 (6 pooled umbilical cord donors) Age = Newborn, Sex = Male, Female Mixed, Race = Black, Other, Caucasian, Caucasian, Black, Caucasian The cells were isolated from donated human tissue after obtaining permission for use in research applications by informed consent or legal authorization.
Reporting on race, ethnicity, or other socially relevant groupings	No reference to race, ethnicity or other social groups is made in the manuscript.
Population characteristics	See above
Recruitment	No recruitment was performed.
Ethics oversight	No ethics oversight body was consulted.

Note that full information on the approval of the study protocol must also be provided in the manuscript.

Field-specific reporting

Please select the one below that is the best fit for your research. If you are not sure, read the appropriate sections before making your selection.

☒ Life sciences ☐ Behavioural & social sciences ☐ Ecological, evolutionary & environmental sciences

For a reference copy of the document with all sections, see nature.com/documents/nr-reporting-summary-flat.pdf

Life sciences study design

All studies must disclose on these points even when the disclosure is negative.

Sample size	No statistical method was used to predetermine sample size.
Data exclusions	No data were excluded.
Replication	Major findings were replicated across the rxrx3 and cpg0016 datasets as well as within three data sources from DepMap (19Q3, 22Q4, and 23Q2).
Randomization	No interventional analyses were performed as a part of this study, so no randomization of samples into experimental groups was used.
Blinding	Blinding is not relevant to this study because there were no interventions applied that could be influenced by experimenters.

Reporting for specific materials, systems and methods

We require information from authors about some types of materials, experimental systems and methods used in many studies. Here, indicate whether each material, system or method listed is relevant to your study. If you are not sure if a list item applies to your research, read the appropriate section before selecting a response.

Materials & experimental systems

n/a	Involved in the study
☒	☐ Antibodies
☐	☒ Eukaryotic cell lines
☒	☐ Palaeontology and archaeology
☒	☐ Animals and other organisms
☒	☐ Clinical data
☒	☐ Dual use research of concern
☒	☐ Plants

Methods

n/a	Involved in the study
☒	☐ ChIP-seq
☒	☐ Flow cytometry
☒	☐ MRI-based neuroimaging

Eukaryotic cell lines

Policy information about cell lines and Sex and Gender in Research

Cell line source(s)	The HUVEC source cells used in the rxrx3 dataset were sourced from two donor pools.<br>1) Lonza Catalog # C2519A Lot 0000662339 Manufacture Date 26-Sept-2017 (3 pooled umbilical cord donors)<br>Age = Newborn, Sex = Male, Male, Male, Race = Caucasian, Caucasian, Caucasian<br>2) Lonza Catalog # C2519A Lot 0000661173 Manufacture Date 22-Sept-2017 (6 pooled umbilical cord donors)<br>Age = Newborn, Sex = Male, Female Mixed, Race = Black, Other, Caucasian, Caucasian, Black, Caucasian<br>The cells were isolated from donated human tissue after obtaining permission for use in research applications by informed consent or legal authorization.
Authentication	Cell lines were only utilized from public data sets (cpg0016 and DepMap).
Mycoplasma contamination	Cell lines were only utilized from public data sets (cpg0016 and DepMap).
Commonly misidentified lines<br>(See ICLAC register)	Cell lines were only utilized from public data sets (cpg0016 and DepMap).
